# Tachycardia-induced unexpected pacemaker behaviour

**DOI:** 10.1007/s12471-014-0607-z

**Published:** 2014-10-16

**Authors:** A. Bőhm, R. G. Kiss, G. Z. Duray

**Affiliations:** Department of Cardiology, Military Hospital, Róbert K körut 44, Budapest, Hungary 1344

**Fig. 1:**
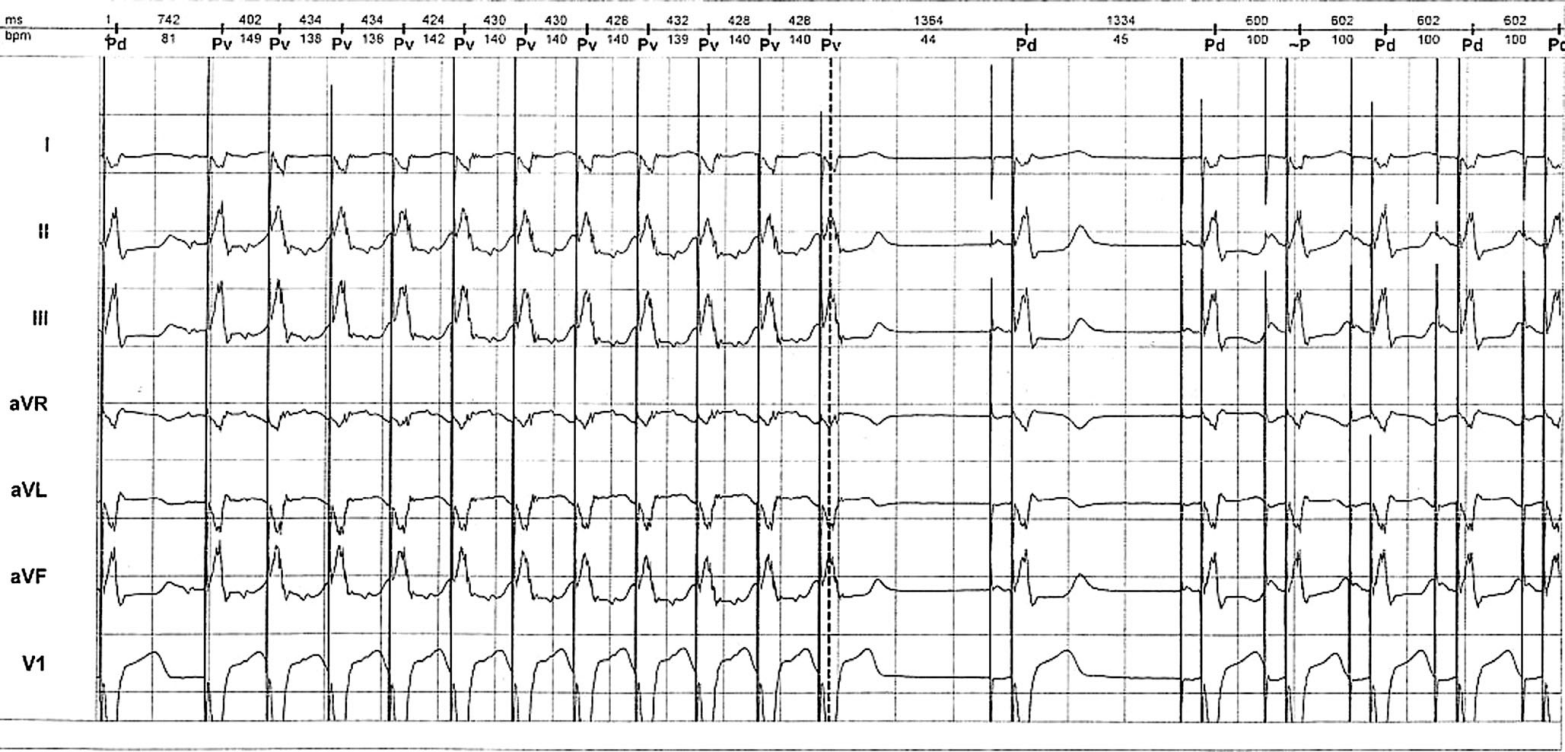
Holter recording of arrhythmia

A Adapta ADDR01 pacemaker was implanted in a 67-year-old female patient due to intermittent complete atrioventricular (AV) block. Appropriate pacemaker operation was detected at regular follow-ups: atrial/ventricular threshold 0.5/1 V, P/R amplitude 2.8/22 mV, lower rate 45 bpm, upper track 140 bpm. Later follow-ups detected pacemaker dependency due to the progression of AV conduction disturbance. Three years after implantation, frequent paroxysmal tachycardias occurred. Holter monitoring recorded the arrhythmia causing the symptoms (Fig. 1).

What is the mechanism of this arrhythmia and unexpected pacemaker operation?

You will find the answer elsewhere in this issue.

